# A study on the hydrodynamic performance of manta ray biomimetic glider under unconstrained six-DOF motion

**DOI:** 10.1371/journal.pone.0241677

**Published:** 2020-11-10

**Authors:** Wen-Hao Cai, Jie-Min Zhan, Ying-Ying Luo

**Affiliations:** 1 Department of Applied Mechanics and Engineering, Sun Yat-Sen University, Guangzhou, China; 2 Institute of Road and Bridge, Guangdong Provincial Academy of Building Research Group Co., Ltd., Guangzhou, China; Coastal Carolina University, UNITED STATES

## Abstract

A manta ray biomimetic glider is designed and studied with both laboratory experiments and numerical simulations with a new dynamic update method called the motion-based zonal mesh update method (MBZMU method) to reveal its hydrodynamic performance. Regarding the experimental study, an ejection gliding experiment is conducted for qualitative verification, and a hydrostatic free-fall experiment is conducted to quantitatively verify the reliability of the corresponding numerical simulation. Regarding the numerical simulation, to reduce the trend of nose-up movement and to obtain a long lasting and stable gliding motion, a series of cases with the center of mass offset forward by different distances and different initial angles of attack have been calculated. The results show that the glider will show the optimal gliding performance when the center of mass is 20mm in front of the center of geometry and the initial attack angle range lies between *A*_0_ = -5° to *A*_0_ = -2.5° at the same time. The optimal gliding distance can reach six times its body length under these circumstances. Furthermore, the stability of the glider is explained from the perspective of Blended-Wing-Body (BWB) configuration.

## 1 Introduction

Manta rays, also known as "devil fish," have evolved for approximately 100 million years, but they still retain the appearance of their ancestors: a flat body in the shape of a diamond. Unlike most spindle-shaped fish, the body of a manta ray is more similar to a giant marine kite. The most typical features of the manta ray’s body are the two large triangular pectoral fins, the wide but short mouth with two head fins, and the tail, which has a smaller triangular-shaped caudal fin but no dorsal fins [1–4]. In addition, the distance between the two pectoral fins of the manta ray is greater than its body length. It is because of this unique physiological structure that manta rays behave more similarly to a bird flying in the ocean [1], which could be found in the attached video in the literature [5].

Due to their unique body structure and swimming form, manta rays have received increasing attention from researchers. Fish et al. [6] studied the hydrodynamic performance of aquatic flapping and found that the most important propulsion was produced from the distal end of the fins, the highest propulsive efficiency was found for Strouhal numbers St = 0.2~0.4. Liu et al. [7] numerically studied the fin thrust producing mechanisms, which is due to the double vortex ring loops shed from the distal part of the fins. Those vortex rings can induce strong backward flow jets which are mainly responsible for the fin thrust generation. Fish et al. [5] measured the turning performance of manta ray from the movies and found that they often make small radius turns due to the rigid body with the highest speed of 67.32 deg/s. These studies are much useful for the design and further research of bio-inspired autonomous underwater vehicles (BAUVs). Scientists have created different kinds of manta robots and have been trying to enhance their propulsion [8–13]. Cai et al. [10] conducted a large number of hydrodynamic experiments and pectoral fin swing propulsion designs of caw-nosed manta rays. The well-known B-2 stealth and strategic bomber of the US military is the most successful example of a manta ray biomimetic aircraft [14].

Braun et al. [15] revealed mantas may use gliding behavior during travel which may conserve energy and maximize movement efficiency. Zhan et al. [16] conducted a hydrodynamic simulation of a manta ray model that was fixed in the uniform flow to investigate the differences in drag coefficients and lift coefficients corresponding to the current speed and attack angles of the manta ray model. Furthermore, three types of ocean dwellers (killer whales, manta rays, and swordfish) with different body parameters were also studied for near-water gliding motion, and the differences in the hydrodynamic performance of the different body shapes were obtained according to a full-scale (2 m) towing experiment of manta rays [17]. Wang et al. [18] studied the hydrodynamic performance of the biomimetic manta ray underwater glider numerically. They found that manta ray has better hydraulic performances with a large angle of attack and a small attitude angle while gliding. However, the above researches are conducted based on the cases of relative motion, i.e., the object (the manta ray model) is fixed in the water, and the uniform flow is a given condition. That means the manta ray model can only move straightly and horizontally at a constant velocity. But in reality, either manta rays or biomimetic aircraft can move freely in three-dimensional space, with two horizontal axes as X and Y, and the vertical axis Z. They can also change orientation between those axes though rotation usually called pitch (face up or down), yaw (turn left or right) and roll. While in this paper, a rigid manta ray biomimetic glider is designed and manufactured by 3D printing technology, and it is able to glide with six-degree-of-freedom (six-DOF) motion once it is ejected at a certain initial velocity and attack angle. What we are interested in is that the change in the speed and trajectory of the glider under a moving posture directly leads to a change in hydrodynamics, and the change of the hydrodynamic force can consequently affect the motion attitude and trajectory. In order to evaluate the gliding ability and hydrodynamic performance of the glider, both experiments and numerical simulations are carried out under different gliding conditions.

## 2 Model and method

### 2.1 Establishment of the glider model

**Fig 1:**
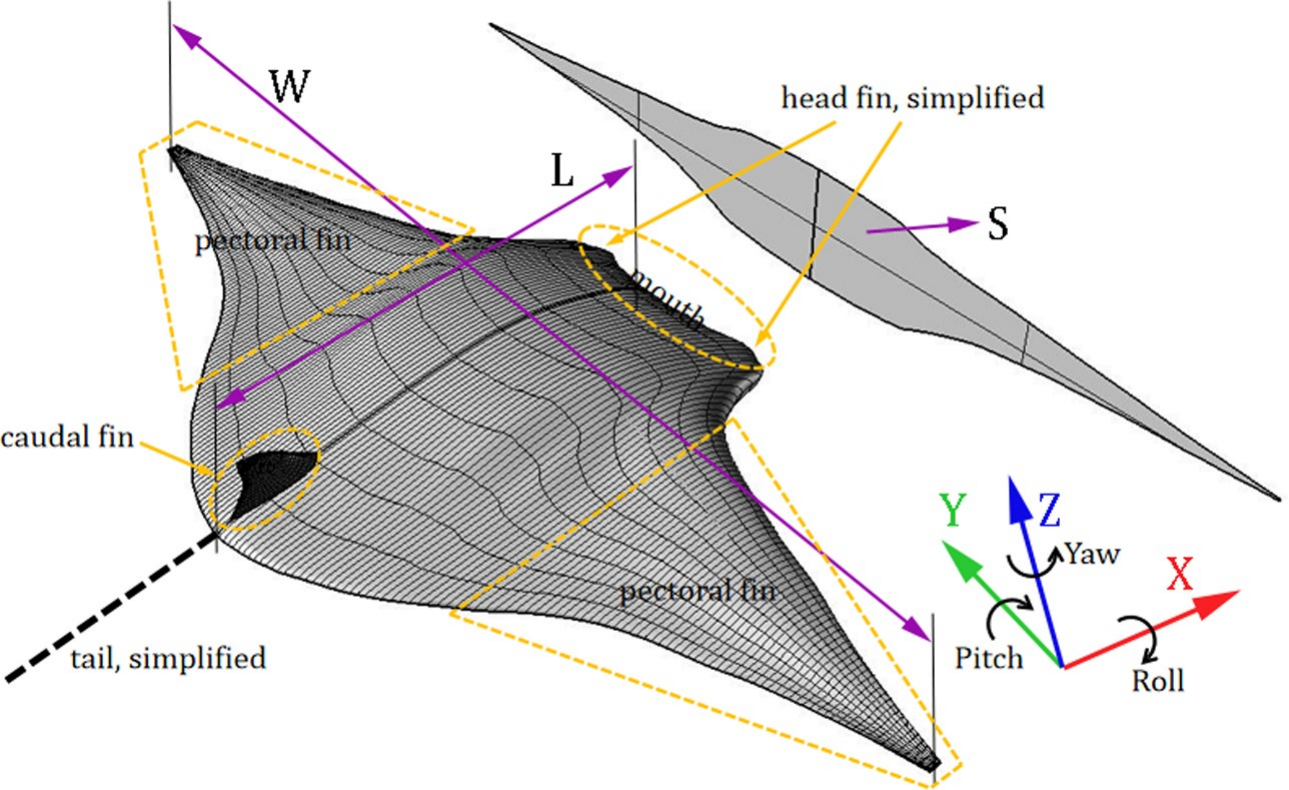
Manta ray biomimetic glider model with a NURBS surface and its computational parameters.

The glider model is established by Non-Uniform Rational B-Spline (NURBS) surface modeling technology. It can easily satisfy the G2 continuity [19], which means that two connected curves on the model not only share the common point, but also have the same tangential direction, the same binormal vector, and the equal curvature at that common point. The parameters of the glider model (both for computation and for experiments) are determined according to the literature [17], where the 3D model is rebuilt based on the 2D photos of real manta rays taken from different angles (the source of the photograph is from open-source website http://fishbase.org). In addition, some essential simplifications and modifications are adopted during model reconstruction; for example, head fins are removed in order to obtain a better streamlined bionic model. More details of the model are as follows: the distance between the two pectoral fins (body width) *W* = 188.5 mm, the body length *L* = 83.0 mm, the projected area of the body on the *YZ* plane *S* = 995.504 mm^2^, the model is manufactured by 3D printing technology with a homogeneous material whose density is 1.141×10^3^ kg/m^3^, and the weight of the model is 84.136 g, as shown in Fig 1.

### 2.2 Mathematical modeling

#### (1) Basic governing equations

The basic governing equation of fluid motion is composed of mass conservation equation and momentum conservation equation, which is the well-known Navier-Stokes equation. For an incompressible homogeneous viscous fluid, the governing equation can be expressed as follows:

$$\begin{array}{c} \nabla \cdot v=0\\ \frac{\partial v}{\partial t}+\left(v\cdot \nabla \right)v=F_{b}-\frac{1}{\rho }\nabla p+v\Delta v \end{array}$$

Where ***v*** is the velocity, *ρ* is the density of fluid, *p* is the pressure, *v* is the kinematic viscosity, ***F***_*b*_ is the body force per unit mass (usually referred to as the acceleration of gravity ***g***), respectively.

#### (2) Turbulence model

In LES model, Navier-Stokes equations are spatially filtered to separate the large scale (resolved scales) eddies from the small scales(residual or sub-grid scales). Filtered governing equations are written in tensor notations as:

$$\begin{array}{c} \frac{\partial \rho }{\partial t}+\frac{\partial \rho {\bar{u}}_{i}}{\partial x_{i}}=0\\ \frac{\partial \left(\rho {\bar{u}}_{i}\right)}{\partial t}+\frac{\partial \left(\rho {\bar{u}}_{i}{\bar{u}}_{j}\right)}{\partial x_{j}}=-\frac{\partial \bar{p}}{\partial x_{i}}+\frac{\partial }{\partial x_{j}}\left[\mu \left(\frac{\partial {\bar{u}}_{i}}{\partial x_{j}}+\frac{\partial {\bar{u}}_{j}}{\partial x_{i}}\right)-\rho \tau_{ij}\right]\\ -\rho \varepsilon_{ikj}\varepsilon_{jlm}\Omega_{k}\Omega_{l}\Omega_{m}-2\rho \varepsilon_{ikj}\Omega_{k}{\bar{u}}_{j} \end{array}$$

where the overbar denotes the resolved quantities, Ω is the rotating speed, and *ε*_*ikj*_ is the Levi–Civita’s alternating tensor. *τ*_*ij*_ is the subgrid-scale (SGS) stress tensor and one of the famous SGS models is proposed by Smagorinsky in accordance with the Boussinesq hypothesis:

$$\tau_{ij}=\frac{1}{3}\delta_{ij}\tau_{kk}-2\nu_{SGS}{\bar{S}}_{ij}$$

where ${\bar{S}}_{ij}$ is the resolved strain rate tensor, *δ*_*ij*_ is the Kronecker delta, and *v*_*SGS*_ is the subgrid kinetic eddy viscosity.

#### (3) Governing equations of Six-DOF motion

The motion of a rigid body could be decomposed to translations along with three directions and rotations around three axes on the basis of barycenter, according to the theory of rigid body motion in classical mechanics. Besides, two coordinate systems, namely the inertial coordinate system and the body coordinate system are usually adopted to describe the six-DOF motion.

The translation of a rigid body can be calculated according to Newton's second law under the inertial system:

$$a_{C}=\frac{1}{m}\sum f_{C}$$

Where ***a***_*C*_ represents the acceleration of barycenter and ***f***_*C*_ represents the forces crossing the barycenter based on the force translation theorem.

Under the inertial system, the relationship between the angular momentum ***L*** of a rigid body and external moment ***M*** can be defined by Euler's second law:

$$M=\frac{dL}{dt}\text{=}\frac{d\left(I\cdot \omega \right)}{dt}$$

Where ***I*** is inertia tensor of rigid body and ***ω*** is the angular velocity.

It is noticed that the inertia tensor of the rigid body in the above formula changes continuously with the movement under the inertia coordinate system, which will increase the difficulty of numerical calculation. Therefore, the rotational motion of a rigid body should be solved under the body coordinate where the inertia tensor can remain unchanged. Considering the material derivative in mathematics, Euler's second law under the body coordinate system is as follows:

$${\left(\frac{d\left(I\cdot \omega \right)}{dt}\right)}_{B}+{\left(\omega \times I\cdot \omega \right)}_{B}=M_{B}$$

where the subscript B represents the body coordinate system.

### 2.3 Dynamic mesh updating method

Because a six-DOF motion of the glider moving in the water will lead to large deformation of the dynamic grid during numerical simulations, a traditional dynamic mesh updating method, such as local remeshing, cannot be used for these cases. In order to make the dynamic mesh updating process more effective and more efficient, we have proposed a new type of dynamic mesh method, named the motion-based zonal mesh update (MBZMU) method, which has successfully been applied in the simulation of the six-DOF motion of a gravity anchor [20, 21], thus, the MBZMU method is adopted for simulation in this paper.

**Fig 2:**
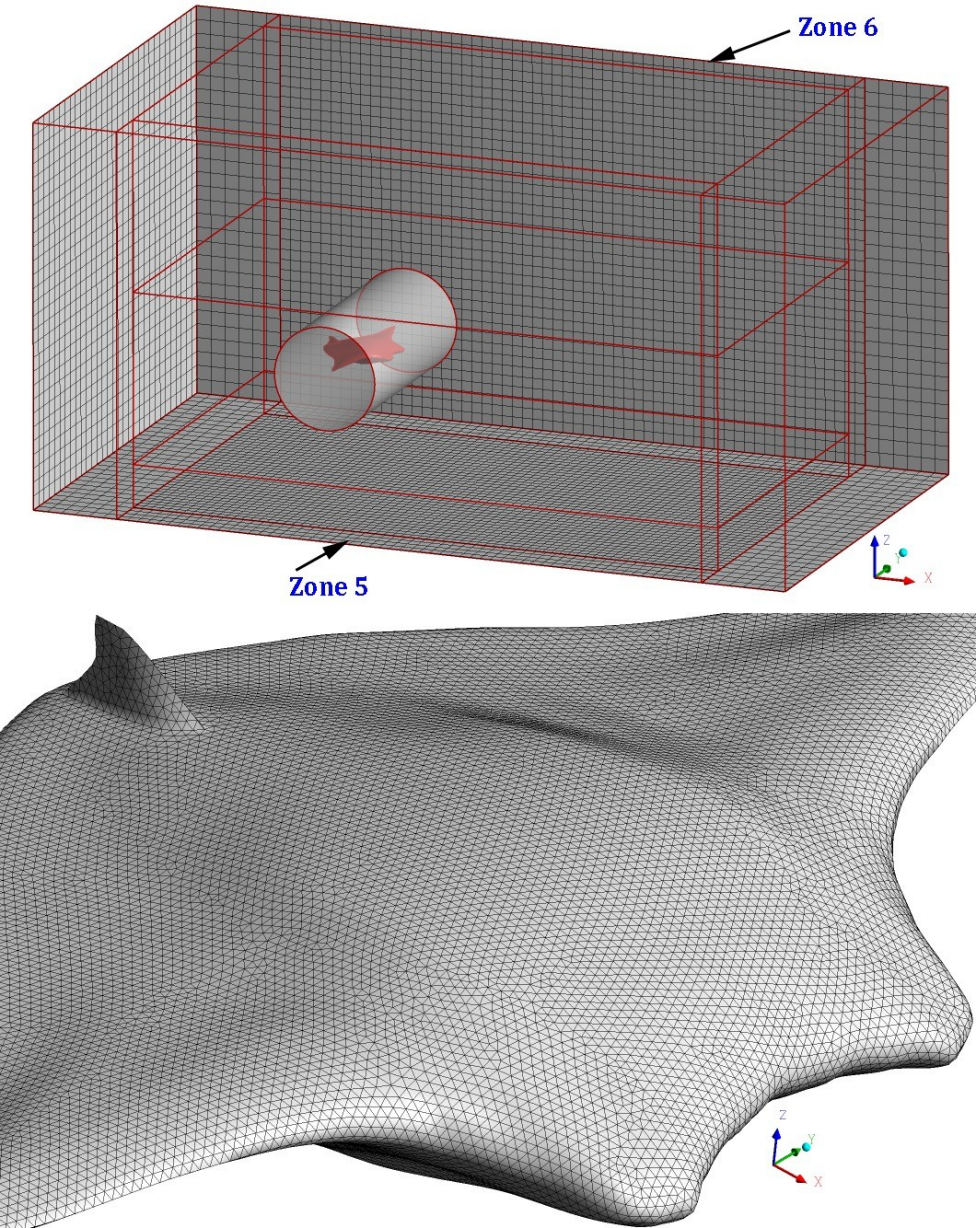
Division of computational domain and grids (upper: Computational domain division based on MBZMU method; lower: Triangular grids on the surface of model).

**Fig 3:**
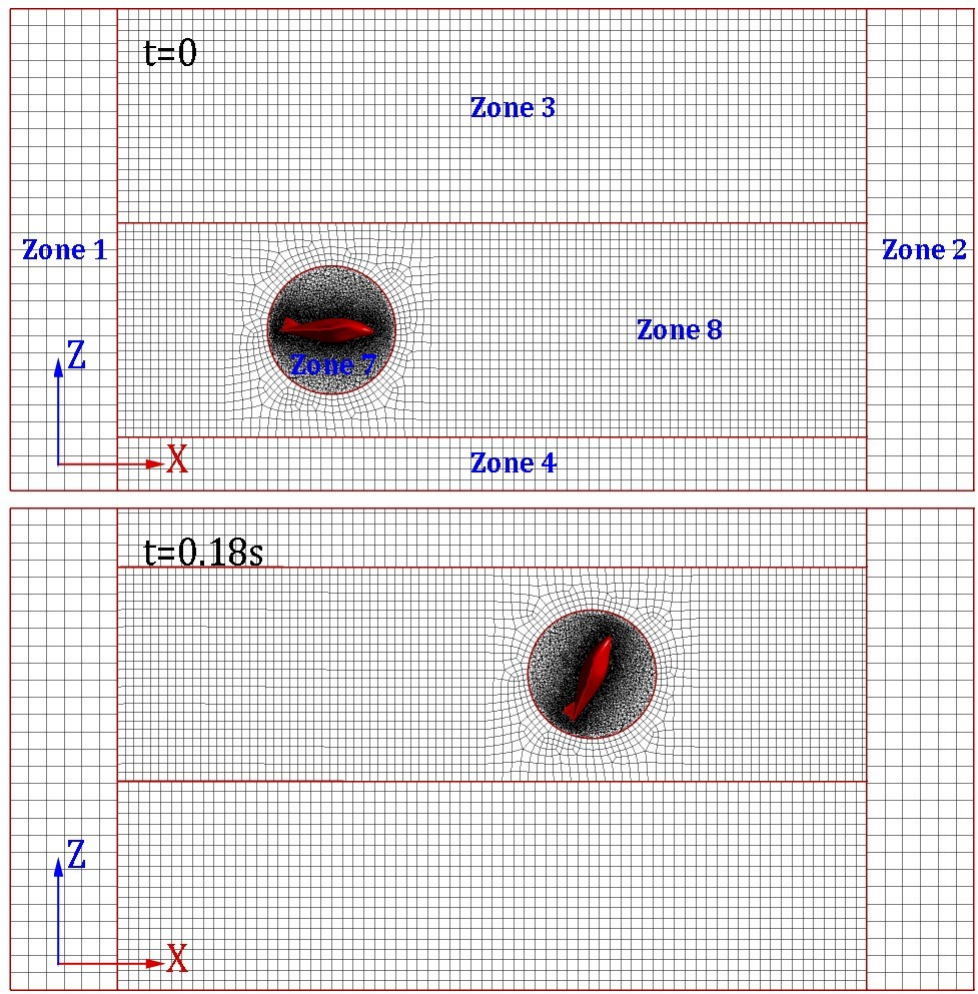
Instantaneous dynamic grids on the symmetry plane.

As described in our previous works [20, 21], the division of the computational domain still takes the same form. i.e., the entire computational domain is successively divided into eight sub-domains numbered 1~8, which is marked in Figs 2 and 3. Sub-domains 5 and 6 are two cuboids located in front and back of the computational domain as shown in Fig 2. It is noted that since the glider's body width is greater than its length, and the model mainly moves in the *XZ* plane. Therefore, the core zone 7 is divided into a cylinder instead of a sphere, thus practice further expands the scope of application of the MBZMU method.

Compared to the dynamic mesh method we used in the past work [22], the MBZMU method can greatly reduce the number of computational grids. Fig 2 shows the division of computational domain and grids generated by ANSYS Gambit. The surface of the model is set as triangular unstructured face grids with a grid size of 1 mm. The core zone 7 containing the model is set as unstructured tetrahedral meshes with a quantity of 2.19 million, while the total computational meshes are only 2.45 million, which shows the superior performance for the MBZMU method in terms of reducing the grid quantity and increasing the grid quality.

The dynamic mesh updating process of MBZMU method can be clearly observed on the symmetry plane, as shown in Fig 3. Because each sub-zone are connected by interfaces with each other, they can move with different degrees of freedom. Namely, zone 8 will move along X-direction and Z-direction with the velocity equal to zone 7, zone 3 and zone 4 will only move along Z-direction. However, zone 1 and zone 2 will remain stationary during the process. The grid split and grid collapse only take place in zone 3, zone 4 and zone 8.

### 2.4 Numerical solver and boundary conditions

The numerical simulation has been conducted in CFD program ANSYS FLUENT, and control of the six-DOF motion of the model is carried out by means of user-defined functions, which is one of the secondary development interface tools provided by ANSYS. Four side faces and underside of the numerical tank are specified as walls, and the top surface is specified as the pressure outlet boundary.

## 3 Experimental study

### 3.1 Ejection gliding experiment

**Fig 4:**
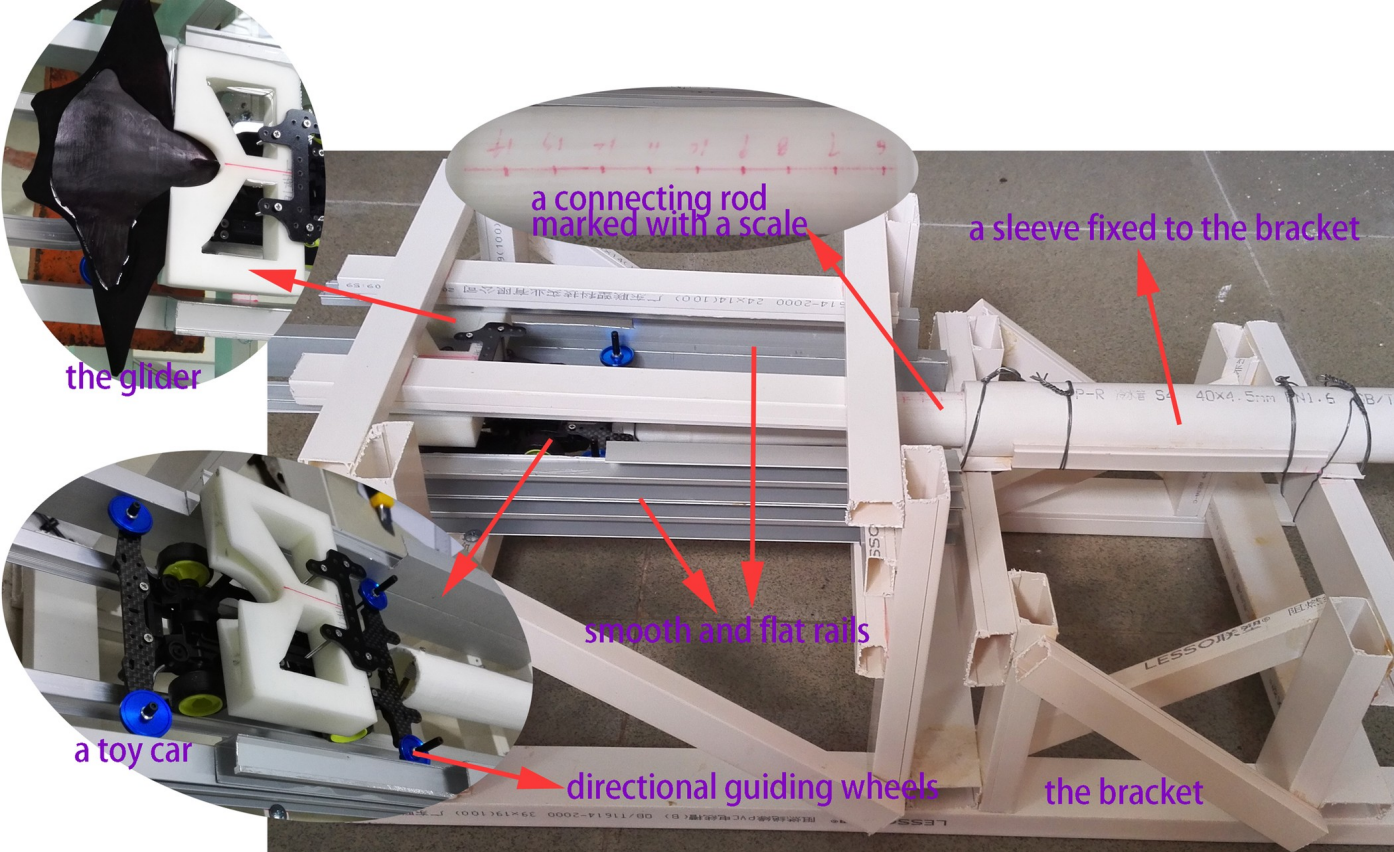
Ejection-gliding experimental device.

The experiment was conducted in a rectangular aquarium (length 2m×width 0.6m×height 0.6m). The glider was created by 3-D printing technology with homogeneous material so that the center of mass is located at the center of geometry. The ejection gliding experimental device is shown in Fig 4. It consists mainly of four parts: a bracket, a launcher, horizontal rails, and the directional guidance device. The composition and functions of each part are described as follows:

1. The ejection bracket made of plastic has a truss structure and is very strong and stable.
2. The launcher consists of a sleeve fixed to the bracket, a spring inside the sleeve, and a connecting rod connected to the spring (the connecting rod is marked with a scale). During the experiment, a suitable external force is applied to compress the spring to the required compression deformation, and then the external force is removed, so the glider model and connecting rod are ejected out from the launcher at a specified speed. The model will separate from the launcher at the moment the spring returns to its original length, then exhibiting free gliding motion.
3. To make the model accelerate slowly during the launching stage, it is necessary that the model has no deviation in the Y and Z directions. Therefore, the ejection device is required to have at least six rails, two of which are at the bottom and two of which are at the top to limit movement in the Z direction, and two rails are on two sides to limit movement in the Y direction.
4. The guidance device is converted from a toy car, which has four wheels on the bottom rails and four directional guiding wheels in contact with the side rails.

Due to this configuration, no matter how fast the ejection speed is, the glider model can smoothly and accurately complete the process of “acceleration-separation-ejection”.

**Fig 5:**
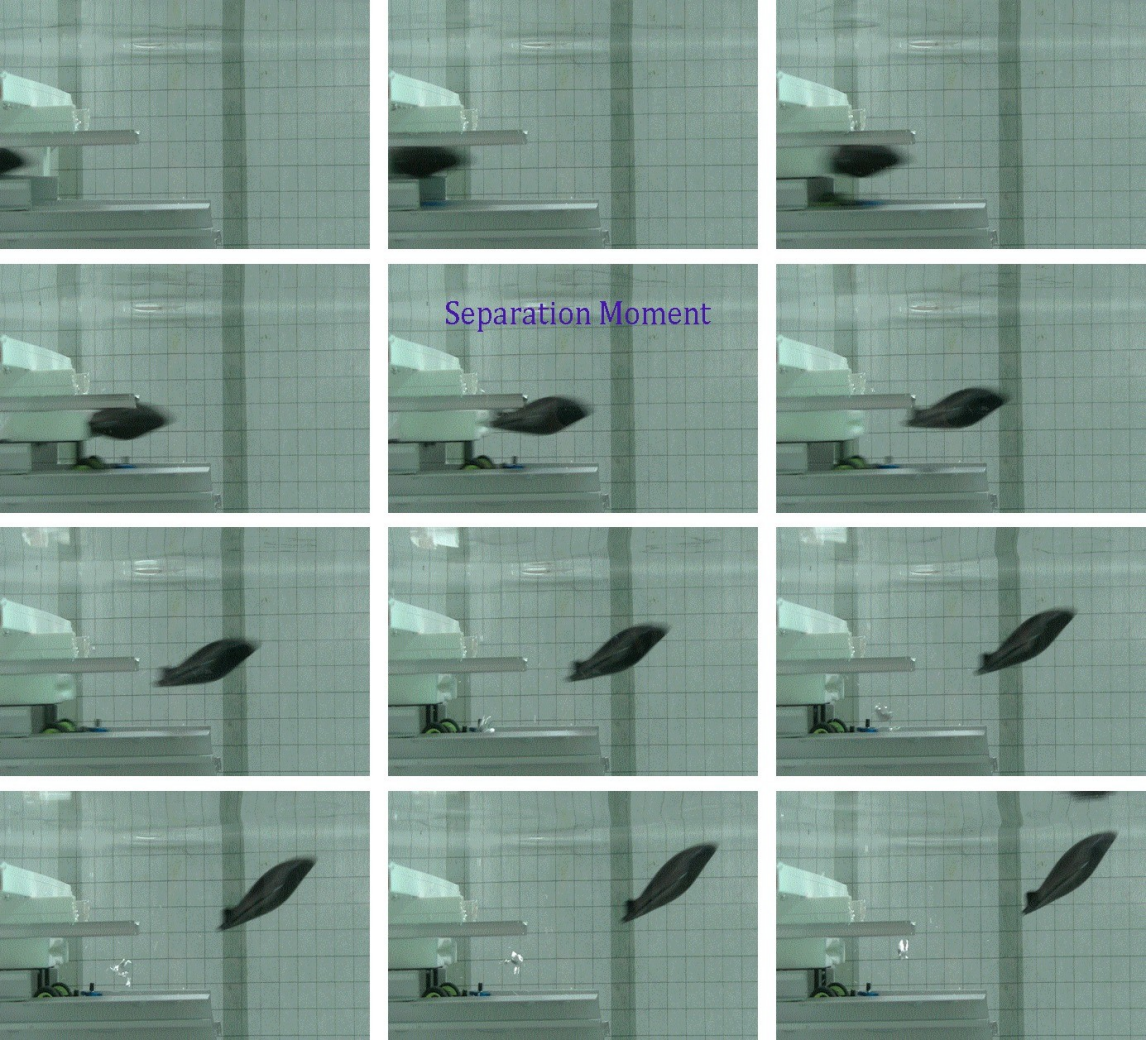
Captured image sequences of ejection-gliding experiments.

Fig 5 shows a series of experimental photographs (captured every 0.02s) for a certain experiment. It can clearly be seen that the model is in a state of continuous acceleration and is always in a stable horizontal posture before separation from the launcher, which indicates that the guidance rails and guidance car play very important roles. Experimental results show that the glider climbs continuously to the water surface, and the deflection angle increases continuously at the same time. We have investigated the gliding stage of human swimmers in previous work to verify the rationality of the traditional optimal instant to initiate underwater leg propulsion [22]. Similar experiments were carried out, and the swimmer model is found to glide almost straight with a small pitch degree [22]. It is noted that the density of the model is higher than that of water, so the reason why the model glides nose-up to the surface of the water is not due to the single factor of buoyancy. The deeper reason might be related to the special shape, the gliding posture and the position of center of mass of the model. This assumption will be verified by combining with the numerical simulation.

In the above experiment, the guidance car produces a strong disturbance in the water during the acceleration process and the separation moment between the model and the launcher. In addition, this disturbance will directly affect the subsequent gliding movement of the glider. Unfortunately, this beginning disturbance is difficult to specify or subtract by means of numerical simulation. To reduce the influence of external factors on the model at the initial moment, we carried out another experiment and simulation in which the model is freely dropped into still water for validation.

### 3.2 Hydrostatic free-fall experiment and validation

**Fig 6:**
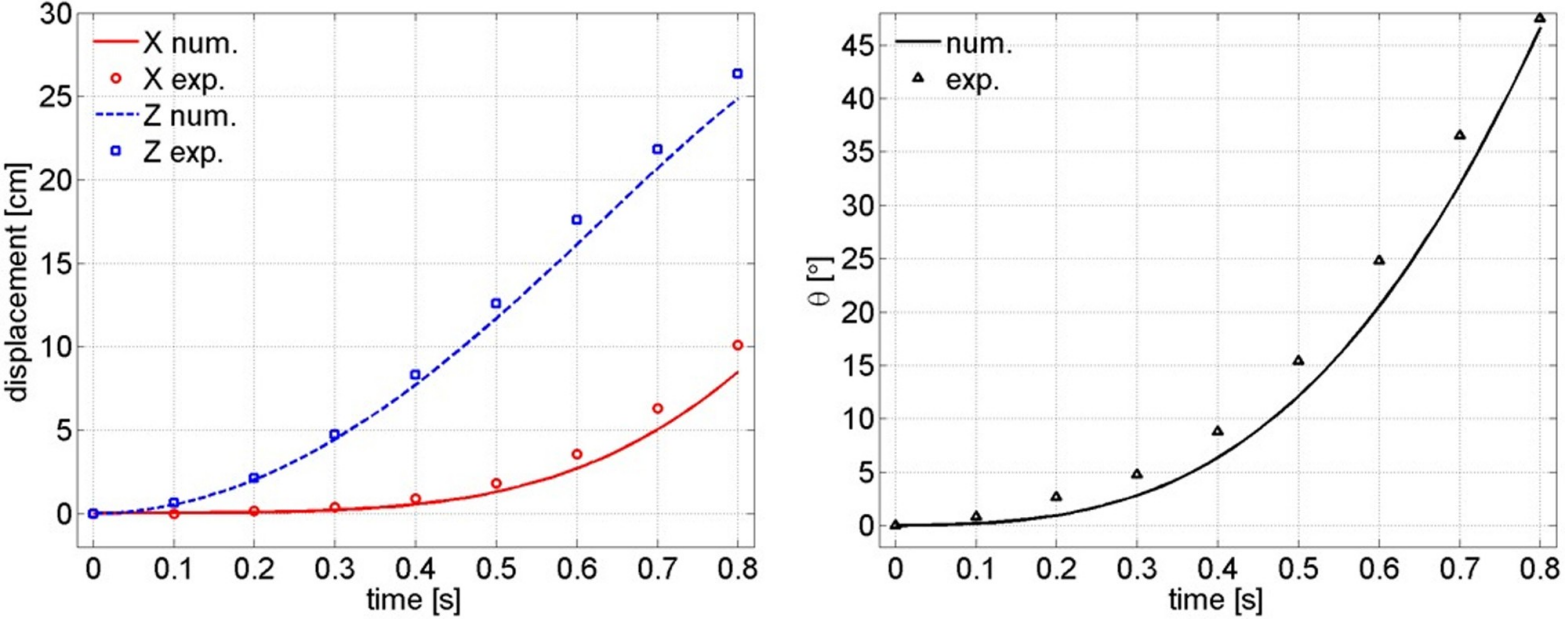
Comparison between hydrostatic free-fall experimental observation and numerical simulation (left: Glider’s displacement; right: The rotation angle *θ*).

In the hydrostatic free-fall experiment, the glider model is set nose-down vertically at the beginning, and then released from a specified position in still water, and a high-speed camera is adopted to record its trajectory and attitude of subsequent movement. Since the experimental model is statically placed at the initial moment, the external factors for the model or the disturbance to water can be almost negligible. This method can effectively deduct the interference of the ejection device on the subsequent movement of the model at the initial moment of the ejection gliding experiment. The experimental observations and corresponding numerical simulation results are compared in Fig 6.

Fig 6 shows that the experimental observations of the displacement and rotation angle *θ* of the model in the hydrostatic free-fall experiment are consistent with the numerical simulation results. In general, the error between the experimental observations and numerical simulations is within a reasonable range, and the rationality of the numerical simulation of hydrostatic free-fall can be verified. On this basis, the hydrodynamic characteristics of the glider’s ejection gliding will be calculated via numerical simulation. Because the calculation grid, the dynamic mesh updating method and the computational configuration are all completely the same as those in the hydrostatic free-fall numerical simulation, so it is sufficient to show that the results of the numerical simulation of the glider’s ejection gliding below are reasonable.

### 4 Numerical simulation

The differences between the lift and drag of a full-scale manta ray model under different attack angles and different water flow velocities are studied in reference [16]. Considering that the biomimetic glider in this paper is a scaled-down model, the initial gliding speed should be set smaller than that in reference [16]. Under the assumption that the glider's center of mass (CoM) coincide with its center of geometry (CoG), two fundamental categories of computational cases are calculated to study the effects of different initial gliding velocities and different attack angles on the trajectory and attitude of the glider.

The first one is that the initial gliding velocities increase from *V*_*0*_ = 0.5 ~ 2.5m/s with an interval of 0.5m/s, and the initial attack angles remain horizontal (i.e., the initial attack angle *A*_*0*_ = 0°). The second one is that the initial attack angles increase from *A*_*0*_ = –7.5°~7.5° with an interval of 2.5°, and the initial gliding velocities remain constant (i.e., the initial gliding speed *V*_*0*_ = 2.5m/s), as shown in Table 1. It is noted that case V2.5 and case A0 are actually the same circumstances, but they are still listed in two lines for convenient description later.

**Table 1:** Two fundamental categories of computational cases with different initial gliding velocities or attack angles.

Case Name	Initial Attack Angle *A*_*0*_(°)	Initial Velocity *V*_*0*_ (m/s)	CoM Position
V0.5	0	0.5	C_0_
V1.0	0	1.0	C_0_
V1.5	0	1.5	C_0_
V2.0	0	2.0	C_0_
V2.5	0	2.5	C_0_
A-7.5	-7.5	2.5	C_0_
A-5.0	-5.0	2.5	C_0_
A-2.5	-2.5	2.5	C_0_
A0	0	2.5	C_0_
A+2.5	2.5	2.5	C_0_
A+5.0	5.0	2.5	C_0_
A+7.5	7.5	2.5	C_0_

**Fig 7:**
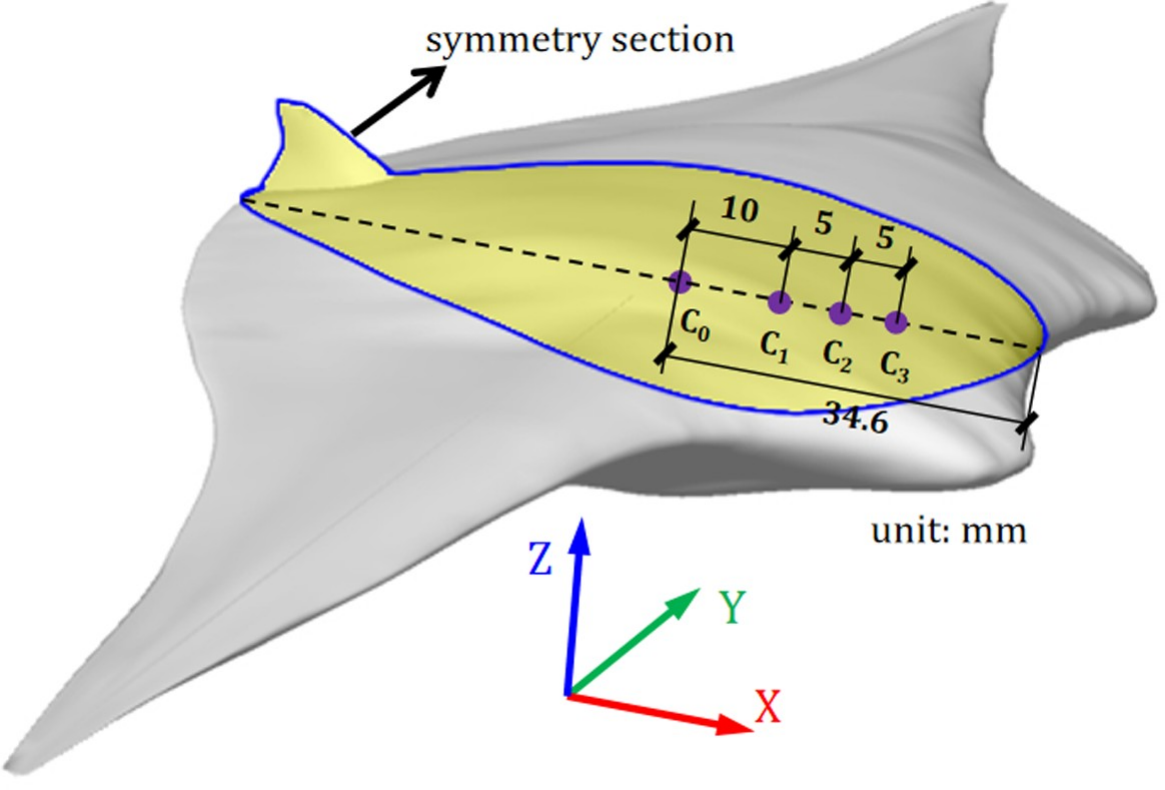
Schematic of cases with different offset forward distances of CoM.

However, The nose-up motions (see Section 5) are observed in above calculations, and the phenomenon is presumed that the pressure center is in front of the CoG. In order to verify this conjecture and to make a better gliding performance of the biomimetic glider, the cases with the CoM offset forward by different distances, Δ = 10*mm*, Δ = 15*mm*, Δ = 20*mm*, are calculated. The initial conditions are list in Table 2 and the CoM positions for different cases are shown in Fig 7, where C_0_ refers to CoG. Moreover, the effects of different attack angles on the trajectory and attitude of the glider with the CoM is situated at C_3_ are studied, which are list in Table 2.

**Table 2:** The initial conditions of cases with different offset forward distances of CoM.

Case Name	Initial Attack Angle *A*_*0*_(°)	Initial Velocity *V*_*0*_ (m/s)	CoM Position
C0	0	2.5	C_0_
C10	0	2.5	C_1_
C15	0	2.5	C_2_
C20	0	2.5	C_3_
OA-7.5	-7.5	2.5	C_3_
OA-5.0	-5.0	2.5	C_3_
OA-2.5	-2.5	2.5	C_3_
OA0	0	2.5	C_3_
OA+2.5	2.5	2.5	C_3_
OA+5.0	5.0	2.5	C_3_
OA+7.5	7.5	2.5	C_3_

## 5 Results and discussion

### 5.1 Trajectory and attitude

#### (1) Cases for different initial gliding velocities

**Fig 8:**
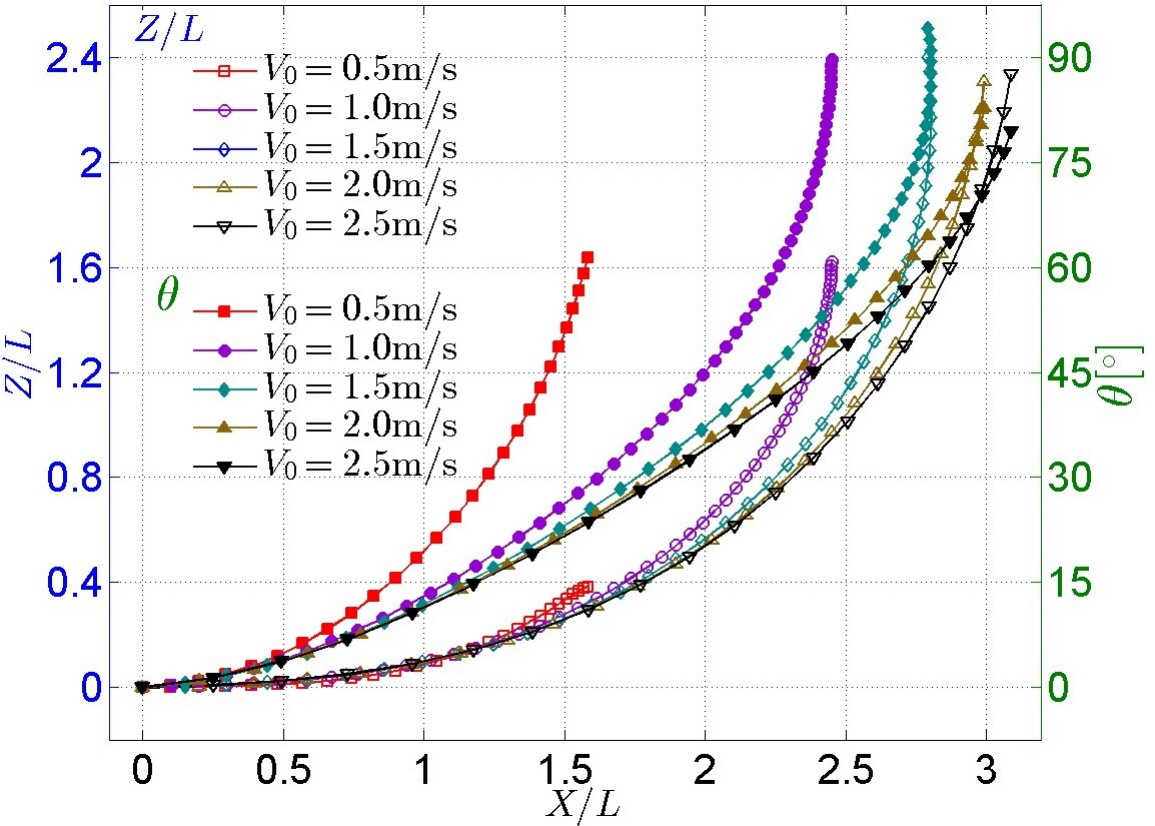
Comparison of trajectory and attitude of model under different initial gliding velocities.

A comparison of the trajectory and attitude of the glider whose CoM is situated at CoG (i.e., the point C_0_ in Fig 7) under different initial gliding velocities is shown in Fig 8, where *X*, *Z*, *L* and *θ* represent the displacement component in the *X* direction, the displacement component in the *Z* direction, the body length of the model, and the angle relative to the initial state, respectively (hereinafter the same). It can be seen from the *Z-X* curve that the trajectories under different initial gliding velocities are almost coincident when the model moves by no more than 1.5 times its body length. The model moves further and higher and the rotation angle *θ* increase as initial gliding velocity *V*_*0*_ increases. The tendency of such an increase is large when *V*_*0*_ is small, but the tendency of such an increase becomes increasingly smaller as *V*_*0*_ increases, which indicates that the gliding trajectory and attitude of the model is affected by the initial gliding velocity *V*_*0*_. However, the dependence and sensitivity of the effect decrease with an increase in *V*_*0*_.

#### (2) Cases for different initial attack angles

**Fig 9:**
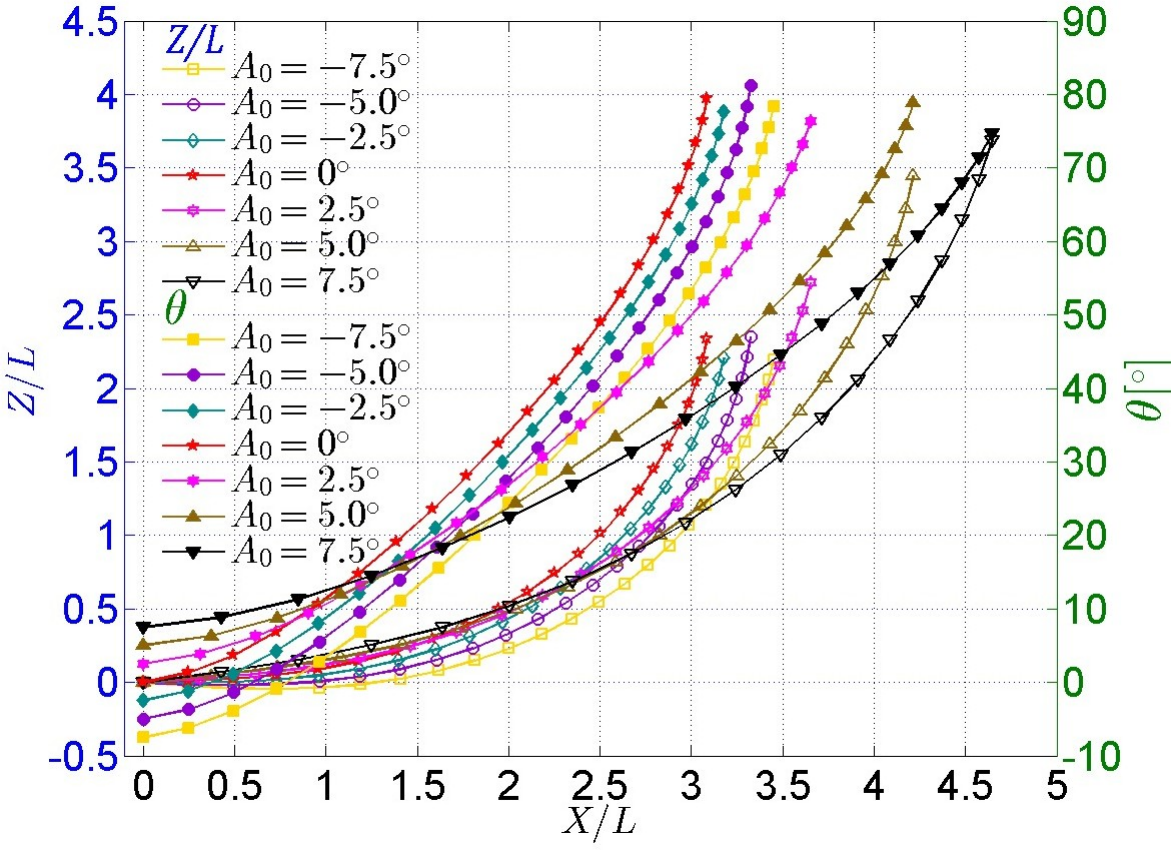
Comparison of trajectory and attitude of the glider under different initial attack angles.

A comparison of the trajectory and attitude of the model under different attack angles is shown in Fig 9. It is obvious that the model under positive initial attack angles (obliquely towards the water surface) goes further and higher than that under negative initial attack angles (obliquely towards the bottom of the tank). When the initial attack angle *A*_*0*_ is positive, the displacement *X* and Z both increase as *A*_*0*_ increases, and the magnitude of the increase is significant. When the initial attack angle *A*_*0*_ is negative, the displacement *X* also increases as *A*_*0*_ increases negatively, but the magnitude of the increase is inconspicuous, while the displacements *Z* are almost the same. Therefore, a conclusion could be reached that the model should have an upward initial attack angle for a greater horizontal gliding distance. The rotation angles of all cases under different initial attack angles almost reach 80° in the end, which indicates the actual rotation angle of the model is independent of the initial attack angle when the initial gliding velocity is constant.

### 5.2 Force on glider model

During the gliding process, the glider model is not only subjected to its own gravity but also to the resistance of the fluid. Generally, the resistance of the fluid *F* could be divided into pressure resistance *F*_*p*_ and viscous resistance *F*_*v*_, namely,

$$F=F_{p}+F_{v}$$

where the pressure resistance *F*_*p*_ is caused by the pressure difference between the front and back of the moving object, which is closely related to the windward area (e.g. the *S* in Fig 5) of the object and the shape of the object [23]. The viscous resistance is due to the viscosity of the fluid and the roughness of the surface of the moving object, and it is also referred to as the frictional resistance.

To reduce the rounding error during CFD simulation, a reference pressure *p*_*r*_ is introduced to normalize the pressure on each computational cell to calculate the pressure resistance. Hence, the net pressure resistance *F*_*p*_ acting on the surface of the model is equal to the sum of the pressure resistance acting on each surface of the cell *p*_*i*_.

$$\begin{array}{c} F_{p}=\sum_{i=1}^{N}\left(p_{i}-p_{r}\right)\sigma_{i}n_{i}\\ =\sum_{i=1}^{N}p_{i}\sigma_{i}n_{i}-p_{r}\sum_{i=1}^{N}\sigma_{i}n_{i} \end{array}$$

where N is the number of triangular face units on the surface of the model, *σ*_*i*_ is the area of a face unit, and *n*_*i*_ is the outside normal vector of the face unit.

Viscous resistance is calculated by Newton's internal friction law:

$$F_{v}=\sum_{i=1}^{N}\mu \frac{\partial v_{i}}{\partial n_{i}}\sigma_{i}\tau_{i}$$

where μ is the dynamic viscosity of water, $\frac{\partial v_{i}}{\partial n_{i}}$ is the velocity gradient along the normal direction at a face unit, and *τ*_*i*_ is the unit tangent vector of the face unit.

**Fig 10:**
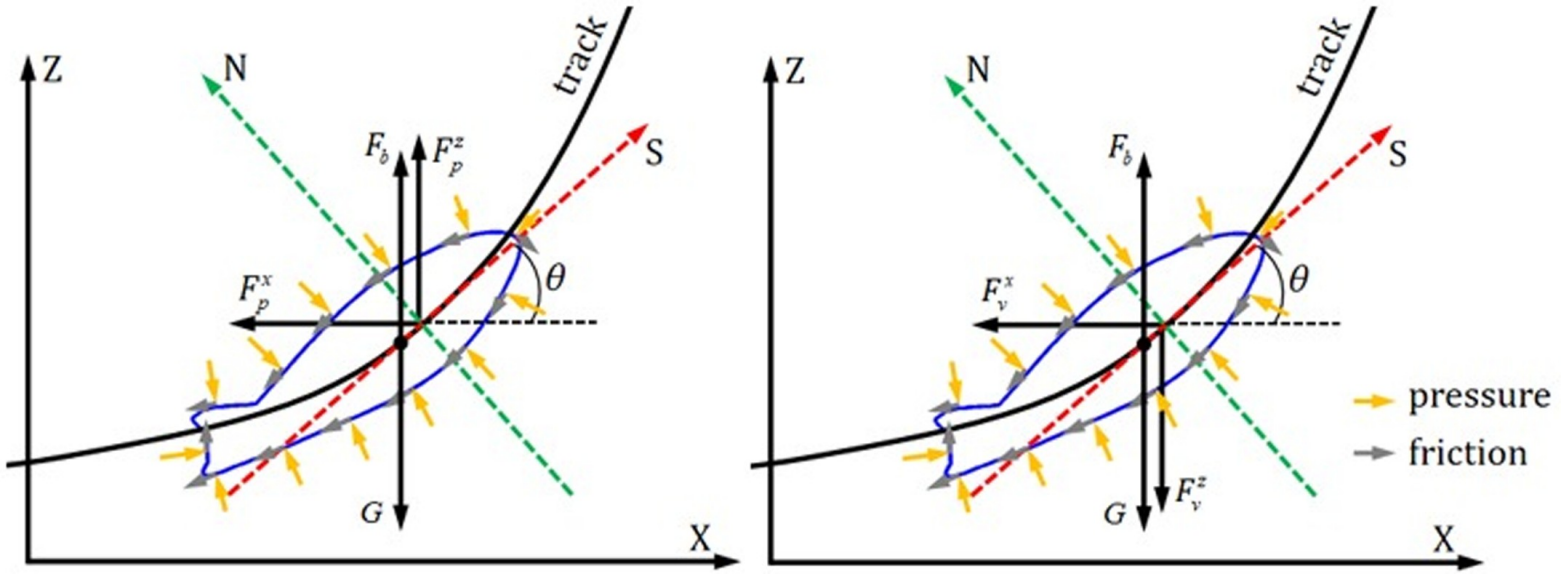
Projections of pressure drag and viscous drag components on the inertial axis to the tangent vector and the normal inner inner normal vector of the trajectory, respectively.

The pressure resistance and the viscous resistance are output according to their components on the inertial coordinate system in the calculation, but the displacement and attitude are always changing during gliding. In order to analyze the hydrodynamic characteristic of the glider, those forces should be decomposed in two directions: the tangent direction of the motion trajectory (i.e., the velocity direction, *S*) and the inner normal direction of the trajectory (*N*), as shown in Fig 10, where *F*_*b*_ and *G* represents the buoyancy and gravity, respectively.

The pressure resistance in the *S* direction and *N* direction ($F_{p}^{s}$ and $F_{p}^{n}$) and viscous resistance in the *S* direction and N direction ($F_{v}^{s}$ and $F_{v}^{n}$) can be obtained by projecting two pressure resistance components $F_{p}^{x}$ and $F_{p}^{z}$, and two viscous resistance components $F_{v}^{x}$ and $F_{v}^{z}$ to the *S* direction and *N* direction, respectively.

$$\begin{array}{l} F_{p}^{s}=-F_{p}^{x}\cos\left(\theta \right)+F_{p}^{z}\sin\left(\theta \right)\\ F_{p}^{n}=F_{p}^{x}\sin\left(\theta \right)+F_{p}^{z}\cos\left(\theta \right)\\ F_{v}^{s}=-F_{v}^{x}\cos\left(\theta \right)-F_{v}^{z}\sin\left(\theta \right)\\ F_{v}^{n}=F_{v}^{x}\sin\left(\theta \right)-F_{v}^{z}\cos\left(\theta \right) \end{array}$$

The sum of the pressure resistance and the viscous resistance in the respective directions of the body coordinate is the total resistance $F_{d}^{s}$ and total lift $F_{l}^{s}$ of the glider on the trajectory, respectively.

$$\begin{array}{l} F_{d}^{s}=F_{p}^{s}+F_{v}^{s}\\ F_{l}^{n}=F_{p}^{n}+F_{v}^{n} \end{array}$$

It should be noted that the above-calculated values can be positive or negative, where a positive value shows that the direction of the component force is the same as the corresponding coordinate axis, and vice versa.

#### (1) Cases for different initial gliding velocities

**Fig 11:**
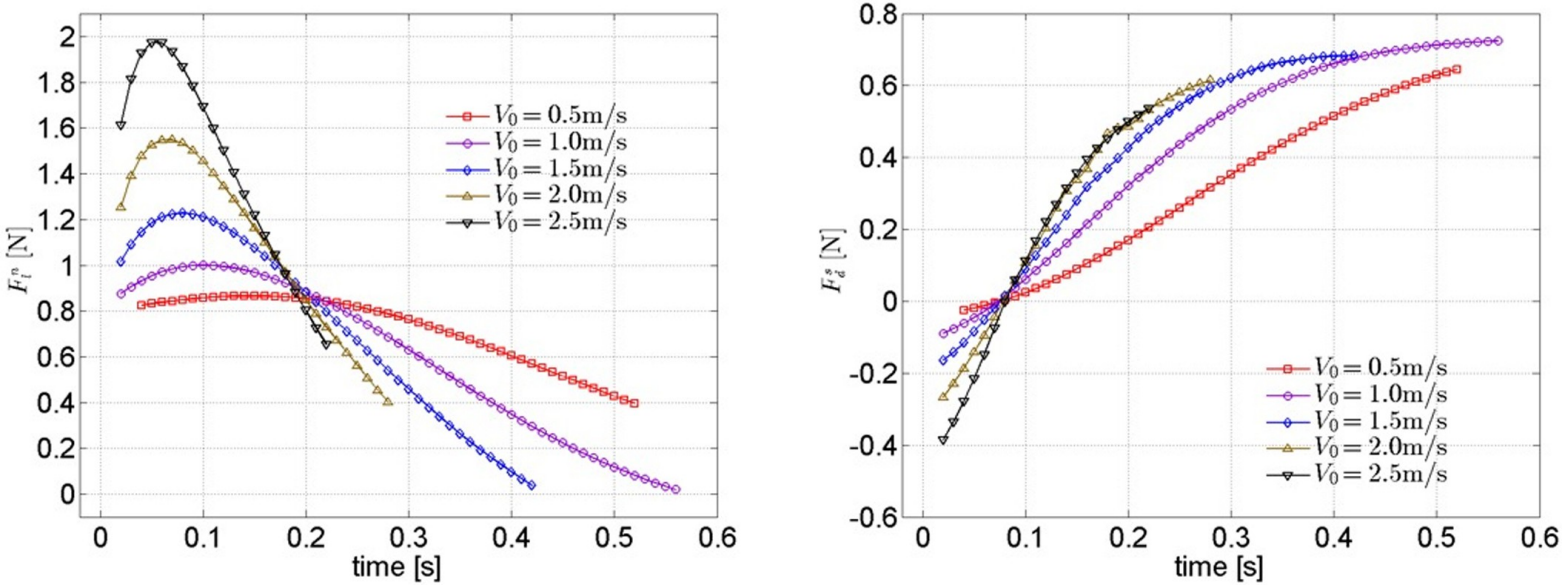
Lift and drag on the trajectory of model under different initial gliding velocities.

The total lift $F_{l}^{s}$ and total drag $F_{d}^{s}$ in the corresponding direction can be obtained by adding the pressure resistance and the viscous resistance in each direction, as shown in Fig 11.

All of the total lifts in the N direction for different cases first increase to a peak then decrease as time goes by ($F_{l}^{n}-t$ curve). Furthermore, the speed of such an increase and decrease become increasingly rapid with an increase in *V*_*0*_. However, the situation is opposite for total drags in the S direction ($F_{d}^{s}-t$ curve). All $F_{d}^{s}$ point in the -S direction and gradually decrease to zero at the beginning of gliding, then they turn to point in the +S direction and gradually increase to a certain stable value (approximately between 0.7 and 0.8 Newton). Moreover, the sensitivity of $F_{d}^{s}$ to the initial gliding velocity *V*_*0*_ decreases with an increase in *V*_*0*_.

Additionally, two special moments can be found in Fig 11: the first special moment is that all of the total drags on the trajectory under different initial gliding velocities *V*_*0*_ decrease to $F_{d}^{s}\approx 0$ when *t* ≈ 0.08s; the second one is that all of the total lifts on the trajectory under different initial gliding velocities *V*_*0*_ decrease to 20304038 when *t* ≈ 0.2s.

#### (2) Cases for different initial attack angles

**Fig 12:**
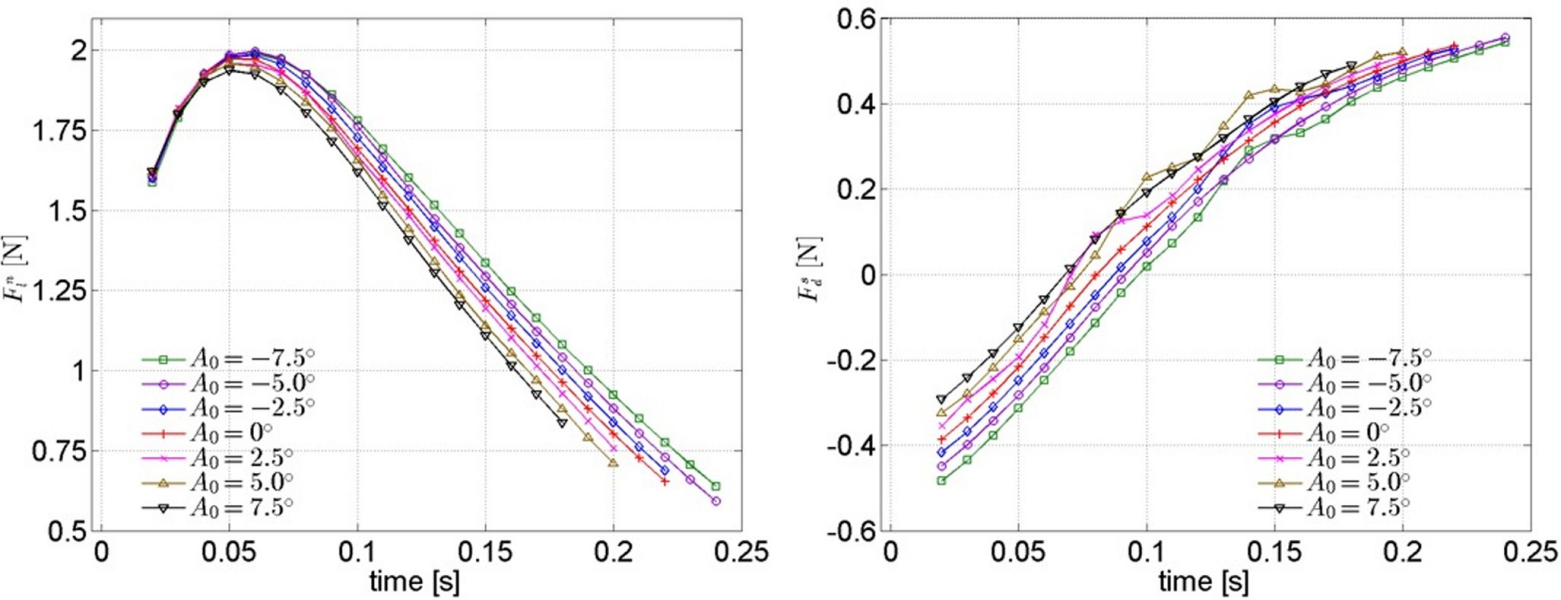
Lift and drag on trajectory of model under different initial attack angles.

For the cases under different initial attack angles, the total lift and total drag are discussed in Fig 12.

All of the lifts for different cases first increase to a peak then decrease as time goes by ($F_{l}^{n}-t$ curve). However, this is not the case for the drags ($F_{d}^{s}-t$ curve). Namely, all of the drags for different cases first decrease to zero (promoting the movement) then gradually increase (hindering the movement) as time goes by. In addition, it can be determined from the $F_{l}^{n}-t$ curve that all of the curves for different cases are almost coincident in approximately *t* < 0.05s, indicating that the total lift on the trajectory is not sensitive to *A*_*0*_ within a very short time after the model starts moving.

### 5.3 The influence of CoM position

Manta rays have a good gliding ability in nature; however, the nose-up motion of the manta-inspired biomimetic glider does not match the above fact. The reason for the situation is speculated that the pressure center of the glider is located in front of the CoM, in order to verify the assumption, several cases with the CoM offset forward by different distances have been studied, as stated before.

**Fig 13:**
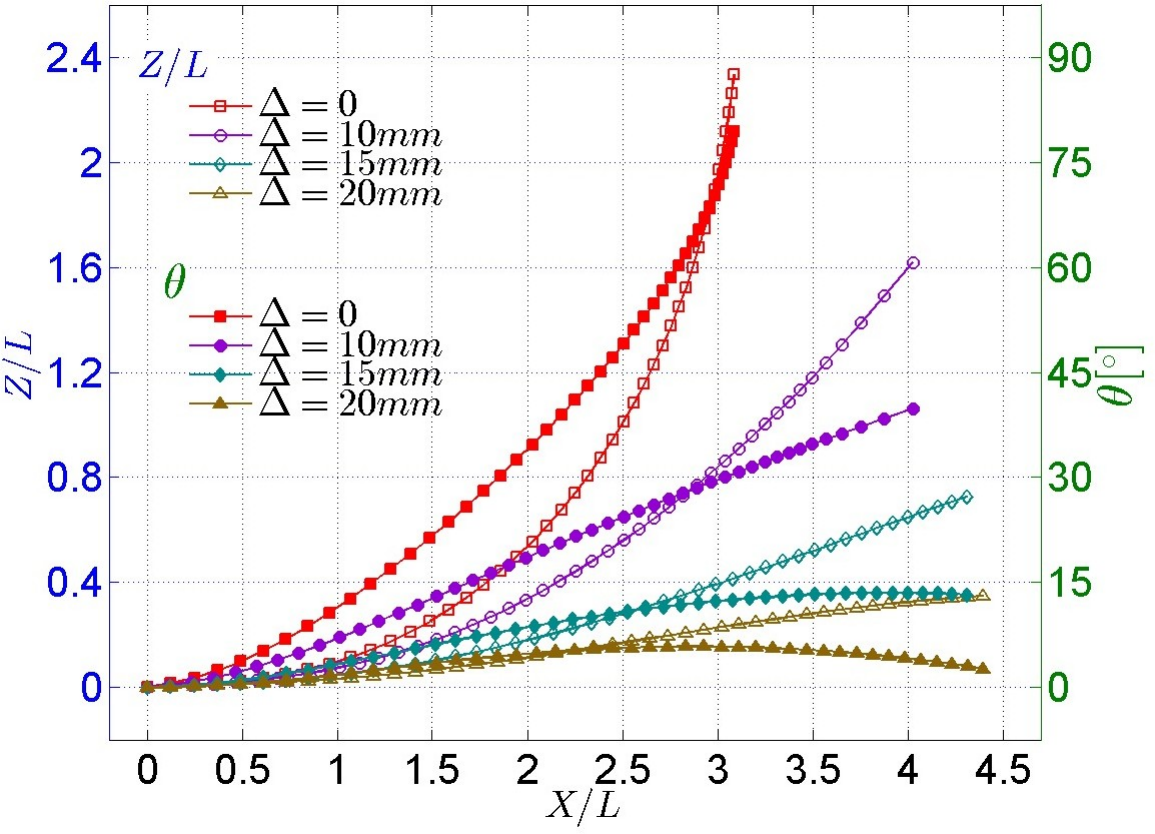
Trajectory and attitude of the cases with different CoM positions.

The trajectory and attitude of the cases with different CoM positions are shown in Fig 13, it can be clearly found that the nose-up motion tendency of the glider get smaller as the CoM is offset forward. When the offset forward distance Δ = 20*mm*, the glider has the longest gliding distance and the smallest vertical distance as well as smallest rotation angle. In other words, the glider will show a good gliding performance if the CoM is designed at an appropriate point.

### 5.4 The gliding performance analysis

As already known in the above section that the glider has a nice gliding performance when CoM offset forward distance Δ = 20*mm*; the gliding performance of different initial angles of attack in this situation is worth studying.

**Fig 14:**
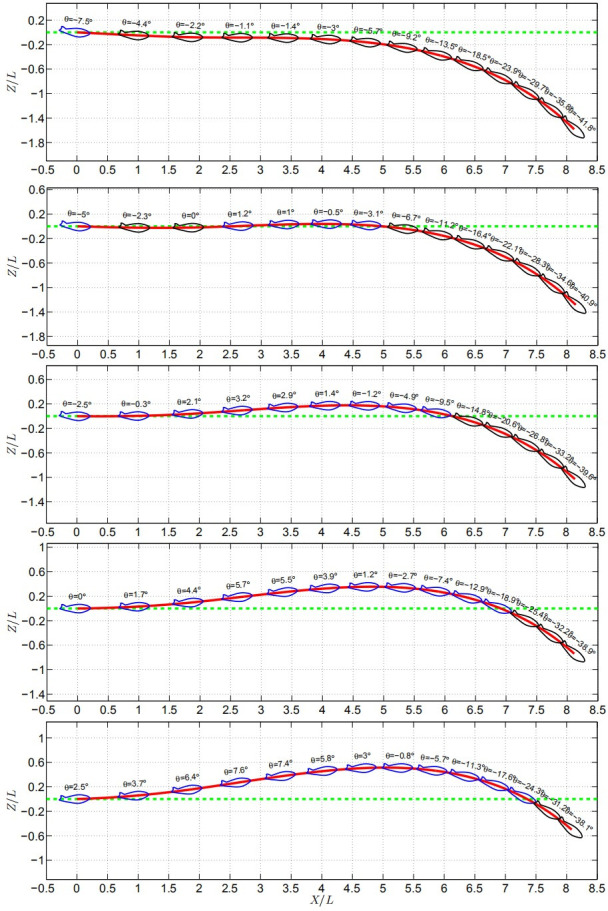
The gliding performance of the glider with different initial attack angles.

The trajectory and attitude of cases with different initial attack angles are shown in Fig 14, where the red line represents glider’s trajectory, the green line represents the horizontal reference line, and the blue line and black line represents the instantaneously captured snapshot of the glider, and the blue/black lines represent the instantaneously captured snapshots of the glider above/below the reference line. The results for the initial attack angle equal to 5 and 7.5 degrees are not shown here, because the trend of nose-up movement becomes more and more intense.

To evaluate the horizontal gliding distance of the glider under different initial attack angles, the distance of two intersections of the horizontal reference line (red) and trajectory (green) is investigated. The results show that the horizontal gliding distance are 0, 5L, 6.2L, 6.8L, 7.4L, 7.7L, 8L with the initial attack angle increasing from -7.5° to 7.5°, respectively. And the maximum vertical distance of nose-up motion are 0, 0.05L, 0.2L, 0.4L, 0.5L, 0.7L, 0.9L, respectively. In other words, the glider has the longest horizontal gliding distance as well as the biggest vertical distance of nose-up motion when initial attack angle *A*_0_ = 7.5°. However, the optimal gliding performance means that the glider should have long enough horizontal distance, a sufficiently small change of the posture (rotation angle), and as little vertical deviation as possible. Therefore, the glider will show the optimal gliding performance when the initial attack angle range lies between *A*_0_ = -5° to *A*_0_ = -2.5° according to the above criterion. The glider will horizontally glide by six times its body length on these circumstances.

### 5.5 Flowfield

It is necessary to analyze the characteristics of the pressure distribution on or near the surface of the gliding model, based on a conclusion reached in the previous analysis that the resistance of the glider is mainly contributed by the differential pressure.

#### (1) Cases for different initial gliding velocities

**Fig 15:**
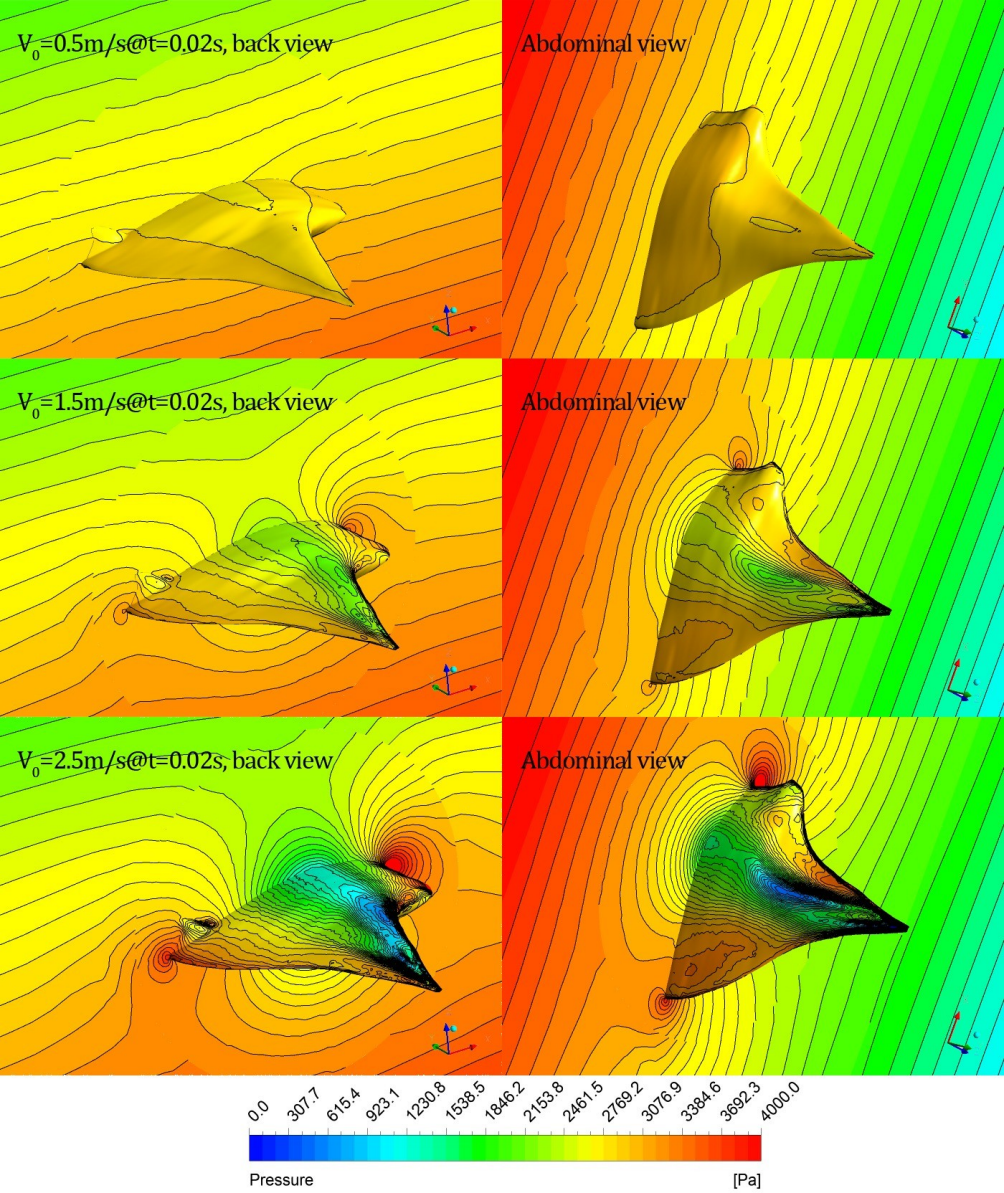
Instantaneous pressure distribution on section *y* = 0 and the surface of the glider under different initial gliding velocities (left: Back view; right: Abdominal view).

The pressure distribution of the symmetrical section *y* = 0 and the surface of the glider under three cases *V*_0_ = 0.5m/s, *V*_0_ = 1.5m/s and *V*_0_ = 2.5m/s are shown in Fig 15, where the instantaneous moment *t* = 0.02*s* and the displayed pressure ranges for three cases are all 0~4000Pa.

For a relatively small initial gliding velocity *V*_0_ = 0.5m/s, the pressure distribution on section *y* = 0 and the surface of the model is still consistent with the hydrostatic pressure distribution, and the contour density on the glider surface is sparse. However, the hydrostatic pressure distribution feature on the section *y* = 0 is gradually destroyed and the contour on the surface of the model becomes denser with an increase in *V*_*0*_, indicating that the pressure gradient increases as *V*_*0*_ increases. There is a high-pressure zone in front of the head and another one behind the tail of the model, and the pressure of the former is larger than that of the latter. There are two low-pressure zones that are nearly equal to one another; one is located on the back of the pectoral fin near the leading edge, and another one is located on the abdomen of the pectoral fin near the symmetrical midline.

Additionally, the pressure in the high-pressure zone increases and the pressure in the low-pressure zone decreases with an increase in *V*_*0*_.

**Fig 16:**
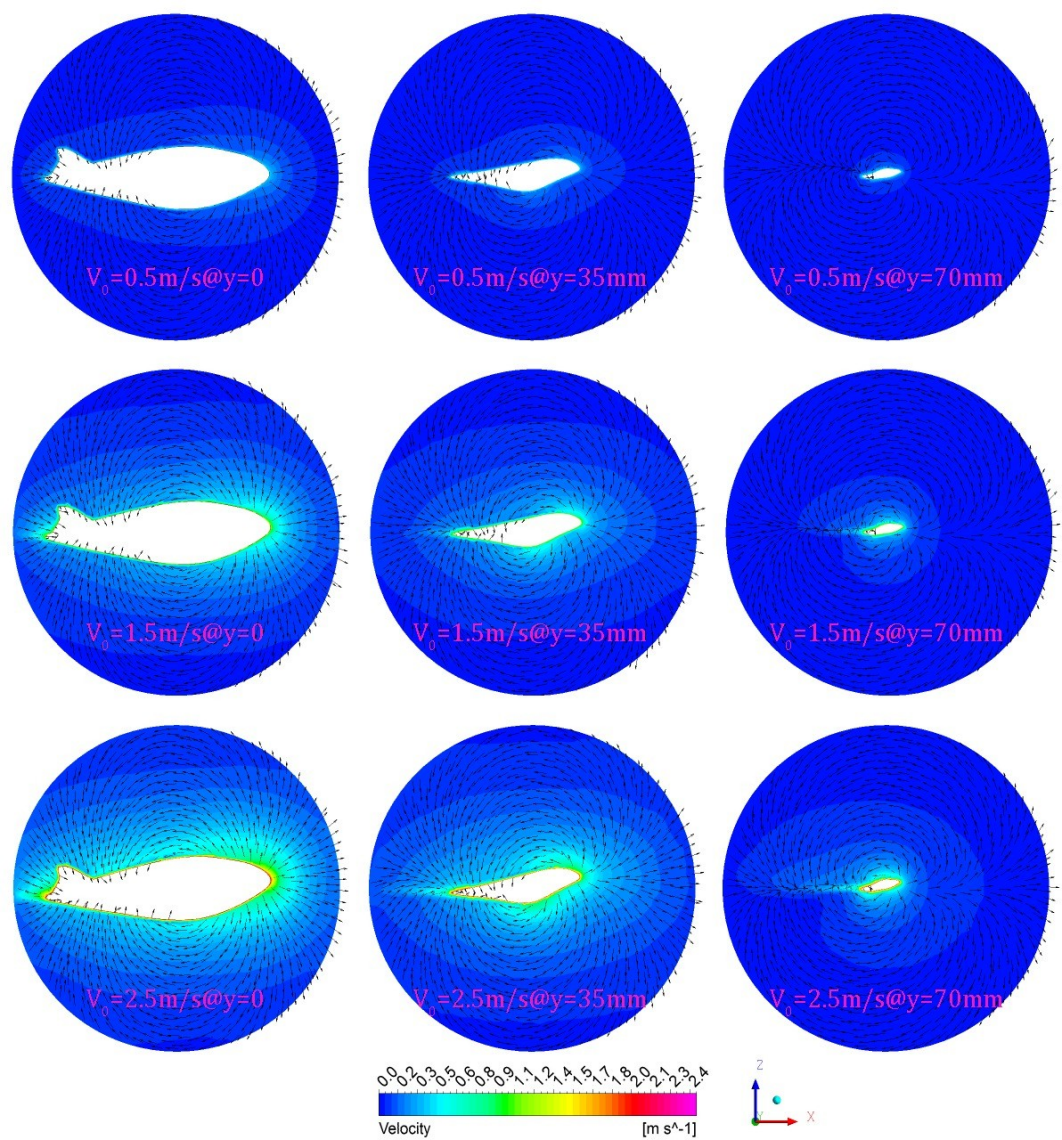
The instantaneous velocity contour and projected vector of the three sections under different initial gliding velocities.

The manta ray biomimetic glider in the present paper is actually a Blended-Wing-Body (BWB) configuration, which is a revolutionary concept for commercial transports and a potential option for future civil aircraft and underwater glider (UG) [24]. Different from the traditional underwater vehicle, the BWB underwater glider merges the traditional fuselage and wing structure into a shape similar to a flying wing, which is able to glide through the water by controlling their buoyancy and converting the lift on wings into propulsive force without a power propulsion system [25]. In order to clearly demonstrate the advantages of the BWB configuration, three different sections (located at XZ-plane, namely, y = 0, y = 35mm and y = 70mm, respectively) are created and the velocity contour of the flow field, as well as the projected normalized velocity vector when t = 0.02s (under case V0.5, V1.5 and V2.5), are shown in Fig 16. It can be seen that the corresponding speed vector diagrams under different initial gliding velocities at different section are similar with each other, namely, the water flow above the back moves counterclockwise, and the water flow below the abdomen moves clockwise.

The above flow field characteristics indicate that the BWB underwater glider has good gliding stability due to the similarity of the transverse (y-direction) flow fields; such stability has also been observed in the ejection gliding experiments (see Fig 5) that the glider has no transverse drift (displacement in y-direction), roll motion or yaw motion after launching. This stability can also explain why the optimized glider has such a long gliding duration.

#### (2) Cases for different initial attack angles

**Fig 17:**
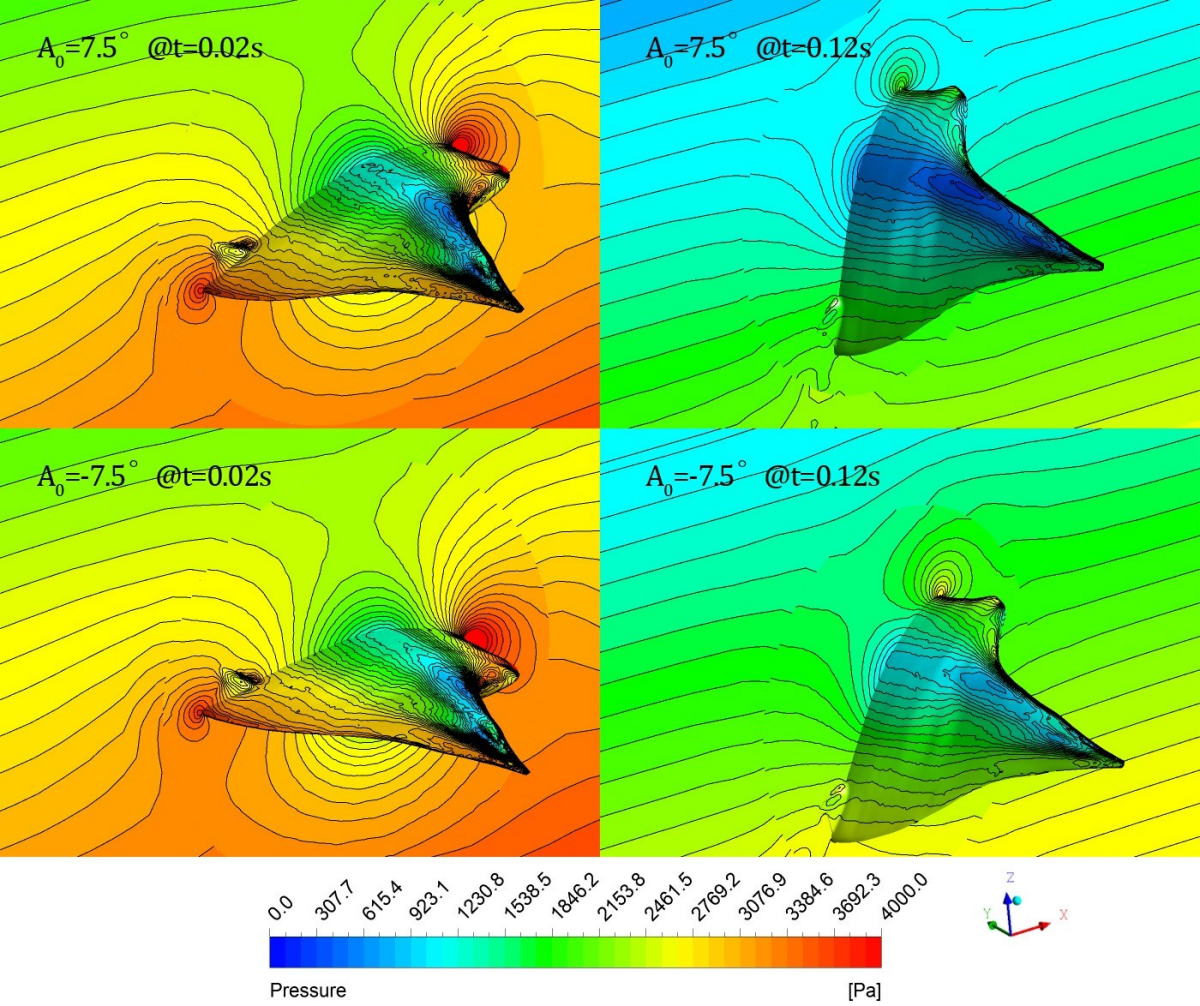
Instantaneous pressure distribution on section *y* = 0 and the surface of the glider under different initial attack angles (left: Back view; right: Abdominal view).

The pressure distribution of the symmetrical section *y* = 0 and the surface of the glider under two cases *A*_0_ = 7.5° and *A*_0_ = -7.5° are shown in Fig 17. Because the initial gliding velocity is the same (2.5 m/s), the pressure distribution for different cases is almost the same, indicating that the pressure distribution in the fluid or on the glider’s surface is closely related to the gliding speed of the glider.

**Fig 18:**
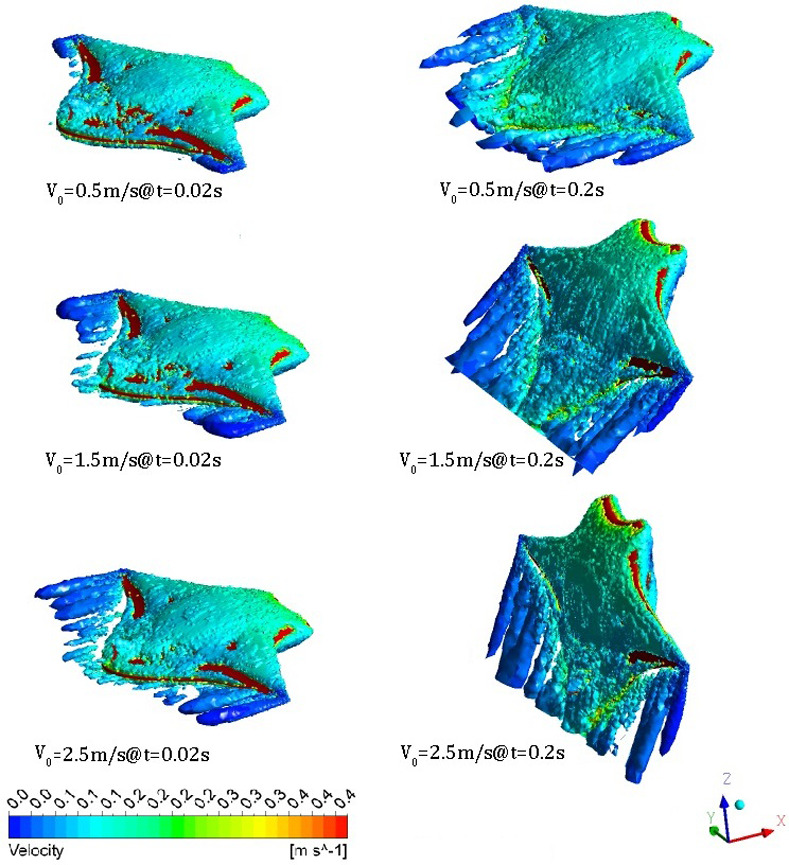
3D instantaneous vortex structures versus time under different initial gliding velocities. (Iso-surface of the swirling strength *W* = 0.004).

It is well known that the vortex structures play important roles for an object moving in a fluid. The three-dimensional instantaneous vortex structures under different initial gliding velocities *V*_0_ = 0.5m/s, *V*_0_ = 1.5m/s and *V*_0_ = 2.5m/s are shown in Fig 18.

It can be seen that the vortex structures for different cases are almost the same. Namely, there are several tube-shaped vortex structures symmetrically distributed behind the pectoral fins at the beginning of gliding, and these tube-shaped vortex structures continuously grow longer and move closer to the rear of the glider as time goes by.

In addition, there is always a relatively large vortex structure on the back and abdomen of the glider surrounding the body, and they will merge with the tail vortex into a large vortex structure as time increases. The difference in the vortex structures between the different initial gliding velocities is performed within the initial gliding stage, i.e., the tube-shaped vortex behind the pectoral fins becomes larger with an increase in *V*_*0*_.

## 6 Conclusion

The hydrodynamic performance of manta ray biomimetic glider under unconstrained six-DOF motion are studied with both laboratory experiment and numerical simulation in the present paper. In order to simulate the unconstrained six-DOF motion of manta ray biomimetic glider, the motion-based zonal mesh update method (MBZMU method), one original dynamic mesh update method proposed by the authors is further improved by replacing a sphere of the core zone into a cylinder. This improvement can not only provide a novel practical technique for the application of the MBZMU method, but also support to study the gliding performance of the glider.

The phenomenon of nose-up motion of the glider has been observed when the center of mass is located at its center of geometry, which has been verified that the pressure center of the glider is located in front of the center of mass by simulating a series of cases with the CoM offset forward by different distances. The results show that the glider will show a good gliding performance if the CoM is designed at an appropriate point, and the position of the most suitable point proved to be 20mm in front of the center of geometry. Furthermore, the gliding performance of different initial angles of attack under the above situation of the center of mass is studied, the numerical show that the glider has the optimal gliding performance when initial attack angle range lies between *A*_0_ = -5° to *A*_0_ = -2.5°, and the glider will horizontally glide by six times its body length on this premise. The above results indicate that it is necessary to design not only a reasonable position of the center of mass but also a reasonable angle of attack for the design of such type of underwater biomimetic glider.

At last, we draw the conclusion that the characteristic of the BWB configuration of the manta ray biomimetic glider is the key factor that it shows good stability in experiments and simulations, and the shape optimization is one of the important research directions of the present biomimetic glider in the future.

## Supporting information

S1 Data (XLSX)
